# Editorial: Epigenetic, metabolic, and transcriptional regulation of immune cell plasticity and functions in cancer and non-cancer diseases

**DOI:** 10.3389/fimmu.2023.1284124

**Published:** 2023-09-25

**Authors:** Yun Dai, Dong Ren, Yafeng He, Huanfa Yi

**Affiliations:** ^1^ Laboratory of Cancer Precision Medicine, the First Hospital of Jilin University, Changchun, Jilin, China; ^2^ Irvine Medical Center, University of California, Irvine, Irvine, CA, United States; ^3^ National Heart, Lung, and Blood Institute, National Institutes of Health, Bethesda, MD, United States; ^4^ Central Laboratory, the First Hospital of Jilin University, Changchun, Jilin, China

**Keywords:** immune cells, epigenetic remodeling, transcriptional reprogramming, cancer, non-cancer disease

High plasticity represents an essential characteristic of innate and adaptive immune cells, which allows their multi-directional transition into diverse phenotypes with different (even opposite) functions (1, 2). The significance of this property is further highlighted by an increasing number of recently emerging new phenotypes of immune cells, particularly involving many malignant and non-malignant disorders (3). Mechanistically, the phenotypic transition occurs via reprogramming of gene expression at the transcriptional level primarily driven by complex and interactive mechanisms involving microenvironment, intracellular signaling, transcription factors, epigenetic remodeling, metabolic rewiring, and post-translational modification (PTM) (1, 4–9).

Transcriptional reprogramming for the phenotypic transition of immune cells is primarily governed via various epigenetic mechanisms, including DNA methylation, histone PTMs, non-coding RNA, RNA modification, etc. (1, 10, 11). DNA methylation usually functions to silence the expression of tumor suppressors in most types of cancer (12). In contrast, many oncogenes are often hypomethylated to promote their expression in tumor cells. Interestingly, multiple differential methylation sites (DMSs) have been found in either the gene itself or the promoter of telomerase reverse transcriptase (TERT), a critical enzyme that controls the length of telomere and is thus considered an oncogene (Lin et al.). Moreover, hypermethylation of these DMSs significantly correlates with TERT expression, infiltration of immune cells [e.g., T cells, T helper 2 (Th2) cells, Treg, CD56^dim^ natural killer (NK) cells, activated dendritic cells (DCs), and B cells], and immune checkpoints (e.g., LAG-3) in triple-negative breast cancer (TNBC) (Lin et al.). Of note, hypermethylation of at least some DMSs is associated with poor overall survival of patients with TNBC (Lin et al.). These findings suggest that TERT promoter hypermethylation may play a role in tumor microenvironment (TME), although the underlying mechanism remains to be explored.

5-methylcytosine (m5C) represents one of the important forms of RNA modification, which occurs on virtually all types of RNA (e.g., mRNA, tRNA, rRNA, lncRNA, and other RNAs) and plays diverse roles in RNA transcription, transportation, and translation (13). Thus, there is no doubt that m5C and its regulatory elements (including “writer”, “eraser”, and “reader”) account for malignant behaviors of both tumor cells and TME involving numerous immune cells (e.g., T cells, B/plasma cells, macrophages, granulocytes, NK cells, DCs, and mast cells) in numerous types of cancer, including solid tumor (e.g., liver, stomach, bladder, prostate, head and neck, breast, pancreas, kidney, and colon and rectum cancer) and hematologic malignancies (e.g., leukemia) (Gu et al.).

Recently, many novel forms of histone and non-histone protein PTMs have been discovered in physiological and/or pathological scenarios (14–17). Among them, lactylation occurs primarily at lysine residues of histones and in some circumstances, also non-histone proteins. Since lactylation is induced by lactate (the end product of glycolysis) (18), it thus represents a key linker between metabolic rewiring (paradigm shift from oxidative phosphorylation to glycolysis or vice versa) and epigenetic remodeling (19, 20). This new PTM may be particularly important in cancer, considering aerobic glycolysis as a metabolic hallmark of cancer known as the Warburg effect (21, 22). Indeed, fast-emerging evidence supports the functional role of lactylation in both tumor cells and TME involving tumor-infiltrating myeloid cells, including tumor-associated macrophages (TAMs), myeloid-derived suppressor cells (MDSCs), and tumor-associated neutrophils (TANs), especially the communication between them (Su et al.). Thus, more reliable targets will become available for anti-tumor epigenetic therapy and immunotherapy soon.

As another protein PTM, ubiquitination is essential for protein turnover via the ubiquitin-proteasome system (UPS) or autophagy, among many other functions, in both normal and malignant cells (23). Consequently, agents targeting UPS (e.g., proteasome inhibitors such as bortezomib) have been extremely successful in the treatment of plasma cell neoplasms like multiple myeloma that relies on a highly-efficient protein turnover machinery to remove abundant useless but harmful immunoglobulin in tumor cells (24). Notably, many E3 ubiquitin ligases (e.g., WWP1/2, SMURF1/2, ITCH, FBXW7, FBXO3/6/21, HECTD1, and ULF1) deubiquitinases (e.g., USP3/5/7/13/14/15/49) that reciprocally regulate ubiquitination are associated with osteoarthritis (Zheng C. et al.), suggesting that this non-inflammatory degenerative joint arthritis may be another disorder caused by deficient protein turnover. In addition to a number of (de)ubiquitination-targeted agents undergoing development (Zheng C. et al.), it is interesting to see whether the FDA-approved proteasome inhibitors would be effective against this type of (de)ubiquitination-deficient disorders like osteoarthritis.

Among various immunotherapies, immune checkpoint inhibition represents a major therapeutic strategy. The most representative one is anti-PD1/PD-L1 monoclonal antibodies (mAbs), which have been approved for the treatment of many types of solid tumors, including lung cancer (25). However, although anti-PD1/PD-L1 mAbs display a striking efficacy, a considerable number of patients with lung cancer have not been benefited (26). To this end, numerous biomarkers have been discovered to pre-select patients who are most likely to benefit from this immunotherapy, including tumor-related markers (e.g., PD-L1 expression, tumor mutation burden, dMMR/MSI, and many tumor-specific genes), peripheral blood-based markers (e.g., ctDNA, immune cells/T cell receptor, and exosomal PD-L1/cytokines) and gut microbiome (Wang et al.). Exhaustion of T cells represents a major hurdle for immunotherapy (27), which may at least in part explain the unsatisfied efficacy of anti-PD1 mAb in most hematologic malignancies like acute myeloid leukemia (AML). In AML patients, accumulation of a subset of severely exhausted T cells (CD28^−^/PD-1^+^/TIGHT^+^) correlates with the presence of minimal residue disease, poor therapeutic response, and short disease-free survival (Huang et al.). Thus, a strategy combining agents targeting such exhausted T cells may improve the efficacy of PD-1 blockade in AML and probably other hematologic malignancies as well. Another approach to enhance the efficacy of immunotherapy is to combine it with radiotherapy, based on a potential mechanism of action involving oxidative stress (e.g., ROS) induced by radiation (Zheng Z. et al.). ROS promotes the release of tumor-associated antigens, which promote infiltration and differentiation of immune cells, modulate the expression of immune checkpoints, and remodel TME (Zheng Z. et al.). Interestingly, expression of certain cell cycle-related genes such as CENPE also correlates with the infiltration of immune cells (e.g., DCs, B cells, T cells, CD4^+^ or CD8^+^ memory T cells, macrophages, and mast cells) at least in some types of cancer (e.g., medulloblastoma) (Fang et al.). However, its functional role in TME, other than tumor cells themselves, and immunotherapy remains to be investigated.

In addition, many other immune checkpoints could also serve as potential targets to fill in the gap left behind. One example is CD47/SIRPα (known as a “don’t eat me” signal for phagocytosis by macrophages) (28). While this checkpoint has been well investigated, its targeted therapy has however not been as successful as anti-PD1/PD-L1 mAbs thus far, largely due to severe adverse events (e.g., anemia, because red blood cells highly express CD47) (29). In this circumstance, many alternative approaches, rather than mAbs, have been attempted to avoid this dark side (30). Targeting glutaminyl-peptide cyclotransferase-like protein (QPCTL) that catalyzes CD47 pyroglutamylation crucial for the binding between CD47 and SIRPα may be a promising approach for the treatment of glioma, in which QPCTL is highly expressed due to DNA hypomethylation and associated with poor outcomes (Liu et al.).

Last, unlike the rapid advance in immunotherapy for cancer, its application in non-cancer diseases lags way behind (31). Among many obstacles, the most important one may be the lack of defined target immune cells in autoimmune or inflammatory diseases, primarily due to the complexity of immune cells with diverse, plastic phenotypes. To this end, recently developed single-cell-analyzing techniques have been doing a really good job of identifying immune cells with different functions (3). For example, single-cell RNA sequencing (scRNAseq) and high-dimensional mass cytometry (CyTOF) have defined a hyper-inflammatory signature in peripheral blood mononuclear cells of patients with Prader-Willi syndrome, with a marked increase in CD16^+^ monocytes that likely drive hyper-inflammatory status of this disease and therefore represent a potential target for immunotherapy (Xu et al.).

Together, further basic, translational, and clinical research with the development and utilization of modern technology would provide much deeper insights into the mechanisms underlying immune cell plasticity (**Figure 1**), leading to the discovery of useful biomarkers or targets for precision medicine against cancer and non-cancer diseases.

**Figure 1:**
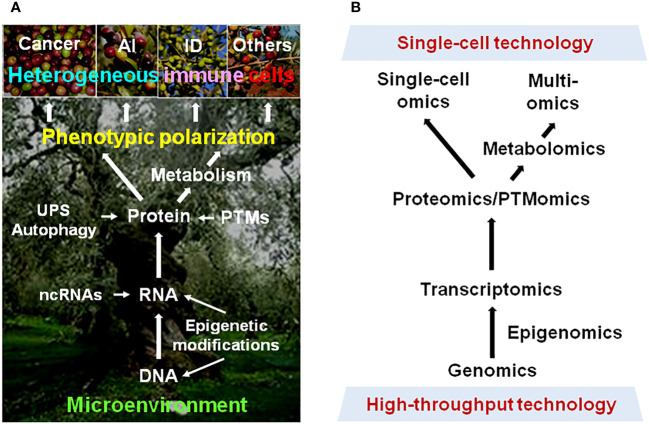
Phenotypic plasticity of immune cells in cancer and non-cancer diseases. **(A)** Multiple dimensions involving the mechanisms underlying the polarization of immune cells into heterogeneous phenotypes with diverse (even opposite) functions in different pathological scenarios. **(B)** Modern approaches for resolving the highly complex mechanisms for plasticity of immune cells in a disease-specific manner, thereby leading to immune microenvironment-targeted therapy for cancer and non-cancer diseases. AI, autoimmune disease; ID, inflammatory disease; UPS, ubiquitin-proteasome system; ncRNA, non-coding RNA; PTM, post-translational modification.

## Author contributions

YD: Conceptualization, Funding acquisition, Resources, Supervision, Visualization, Writing – original draft, Writing – review & editing. DR: Conceptualization, Writing – review & editing. YH: Conceptualization, Writing – review & editing. HY: Conceptualization, Resources, Writing – review & editing.
